# The Birth of a Ghost Star

**DOI:** 10.3390/e27040412

**Published:** 2025-04-11

**Authors:** Luis Herrera, Alicia Di Prisco, Justo Ospino

**Affiliations:** 1 Instituto Universitario de Física Fundamental y Matemáticas, Universidad de Salamanca, 37007 Salamanca, Spain; 2 Escuela de Física, Facultad de Ciencias, Universidad Central de Venezuela, Caracas 1050, Venezuela; alicia.diprisco@ucv.ve; 3 Departamento de Matemática Aplicada and Instituto Universitario de Física Fundamental y Matemáticas, Universidad de Salamanca, 37007 Salamanca, Spain; j.ospino@usal.es

**Keywords:** relativistic fluids, interior solutions, spherically symmetric sources, 04.40.-b, 04.40.Nr, 04.40.Dg

## Abstract

We present a model of an evolving spherically symmetric dissipative self-gravitating fluid distribution which tends asymptotically to a ghost star, meaning that the end state of such a system corresponds to a static fluid distribution with a vanishing total mass and an energy density distribution which is negative in some regions of the fluid. The model was inspired by a solution representing a fluid evolving quasi-homologously and with a vanishing complexity factor. However, in order to satisfy the asymptotic behavior mentioned above, the starting solution had to be modified, as a consequence of which the resulting model only satisfies the two previously mentioned conditions asymptotically. Additionally, a condition on the variation in the infinitesimal proper radial distance between two neighboring points per unit of proper time was imposed, which implies the presence of a cavity surrounding the center. Putting together all these conditions, we were able to obtain an analytical model depicting the emergence of a ghost star. Some potential observational consequences of this phenomenon are briefly discussed in the last section.

## 1. Introduction

In a recent paper [1], the concept of a ghost star was introduced and studied in detail. Such a concept, inspired by the early ideas of Zeldovich [2,3], describes fluid distributions which do not produce a gravitational field outside their boundary surface (i.e., their total mass vanishes). In order to achieve the vanishing of the total mass (for a non-trivial fluid distribution), one must assume the existence of some regions within the fluid sphere endowed with a negative energy density. Some examples of this kind of fluid distribution may be found in [1,4,5] (see also [6] for more recent developments).

More recently, we have studied solutions which either correspond to the adiabatic evolution of a ghost star or describe the evolution of fluid distributions which attain a ghost star status momentarily at some point in their existences, abandoning such a state immediately afterward [7].

It should be stressed that the term “Ghost stars” comes from an analogy with some Einstein–Dirac neutrinos (named ghost neutrinos) which do not produce a gravitational field but are still characterized by a non-vanishing current density [8,9,10]. Thus, any confusion regarding the use of the same terminology in quantum field theory should be dismissed.

However, neither of the models exhibited in the references above describe solutions leading asymptotically (as t→∞) to a ghost star.

It is the purpose of this work to present a model of an evolving fluid distribution describing the emergence of a ghost star as the end point of its evolution.

In order to obtain our model, we initially started by imposing three conditions:

- The vanishing of the complexity factor [11,12];
- Quasi-homologous evolution (QH) [13];
- The variation in the infinitesimal proper radial distance between two neighboring points per unit of proper time vanishes [14].

The first two conditions have been shown to be useful in the description of the structure and evolution of self-gravitating fluids. The second one represents a generalization of the well-known homologous evolution in Newtonian hydrodynamics [15,16,17].

The third condition implies the existence of a cavity surrounding the center and therefore appears to be a useful tool for the modeling of cosmic voids [18,19].

Notwithstanding, we resorted to using the above conditions for purely heuristic reasons, their physical interest being, in the context of this work, a fact of secondary relevance.

The solution obtained under the three conditions above (hereafter referred to as the “primeval solution”) does not satisfy the asymptotic conditions required to obtain a static ghost star as the end point of the evolution. Accordingly, we modified this solution in order to satisfy the conditions ensuring the formation of a ghost star.

The final solution matches smoothly on the external boundary surface with the Minkowski spacetime as t→∞. On the other hand, matching conditions are not satisfied on the boundary surface delimiting the fluid distribution from the inside (not even asymptotically); accordingly, we have a thin shell on that surface.

The physical properties of the model will be analyzed in detail and the characteristics of the ghost star appearing at the end of the evolution will be discussed.

## 2. The General Setup of the Problem: Notation, Variables and Equations

We consider a spherically symmetric distribution of a fluid, which is bounded from the outside by a spherical surface, $\Sigma^{\left(e\right)}$, and since we shall assume there is a cavity surrounding the center, the fluid is also bounded from inside by a spherical surface, $\Sigma^{\left(i\right)}$. The matter content consists of a locally anisotropic fluid (unequal principal stresses) undergoing dissipation in the form of heat flow (diffusion approximation).

Thus, in comoving coordinates, the general line element may be written as

(1) $$ds^{2}=-A^{2}dt^{2}+B^{2}dr^{2}+R^{2}\left(d\theta^{2}+\sin^{2}\theta d\phi^{2}\right),$$

where the functions A,B and *R* depend on *t* and *r*.

The energy–momentum tensor takes the form

(2) $$\begin{array}{ccc} T_{\alpha \beta } & = & \left(\mu +P_{⊥}\right)V_{\alpha }V_{\beta }+P_{⊥}g_{\alpha \beta }+\left(P_{r}-P_{⊥}\right)\chi_{\alpha }\chi_{\beta }\\ & + & q_{\alpha }V_{\beta }+V_{\alpha }q_{\beta }, \end{array}$$

where μ is the energy density, $P_{r}$ the radial pressure, $P_{⊥}$ the tangential pressure, $q^{\alpha }$ the heat flux, $V^{\alpha }$ the four velocity of the fluid, and $\chi^{\alpha }$ a unit four vector along the radial direction. These quantities satisfy

(3) $$\begin{array}{c} V^{\alpha }V_{\alpha }=-1,\;\;V^{\alpha }q_{\alpha }=0,\;\;\chi^{\alpha }\chi_{\alpha }=1,\;\;\chi^{\alpha }V_{\alpha }=0. \end{array}$$

It will be convenient to express the energy–momentum tensor (2) in the equivalent (canonical) form,

(4) $$T_{\alpha \beta }=\mu V_{\alpha }V_{\beta }+Ph_{\alpha \beta }+\Pi_{\alpha \beta }+q\left(V_{\alpha }\chi_{\beta }+\chi_{\alpha }V_{\beta }\right),$$

with

$$P=\frac{P_{r}+2P_{⊥}}{3},\;h_{\alpha \beta }=g_{\alpha \beta }+V_{\alpha }V_{\beta },$$

$$\Pi_{\alpha \beta }=\Pi \left(\chi_{\alpha }\chi_{\beta }-\frac{1}{3}h_{\alpha \beta }\right),\;\Pi =P_{r}-P_{⊥}.$$

Since we are considering comoving observers, we have

(5) $$\begin{array}{ccc} V^{\alpha } & = & A^{-1}\delta_{0}^{\alpha },\;\;q^{\alpha }=qB^{-1}\delta_{1}^{\alpha },\;\;\chi^{\alpha }=B^{-1}\delta_{1}^{\alpha }. \end{array}$$

It is worth noticing that we do not explicitly add bulk or shear viscosity to the system because they can be trivially absorbed into the radial and tangential pressures, $P_{r}$ and $P_{⊥}$, of the collapsing fluid (in Π). Also, we do not explicitly introduce dissipation in the free-streaming approximation since it can be absorbed into $\mu ,P_{r}$ and *q*.

### 2.1. Einstein Equations

Einstein’s field equations for the interior spacetime (1) are given by

(6) $$G_{\alpha \beta }=8\pi T_{\alpha \beta }.$$

The non-null components of (6), along with (1) and (2), read as

(7) $$\begin{array}{c} 8\pi T_{00}=8\pi \mu A^{2}=\left(2\frac{\dot{B}}{B}+\frac{\dot{R}}{R}\right)\frac{\dot{R}}{R}-{\left(\frac{A}{B}\right)}^{2}\left[2\frac{R^{\prime \prime }}{R}+{\left(\frac{R^{\prime }}{R}\right)}^{2}-2\frac{B^{\prime }}{B}\frac{R^{\prime }}{R}-{\left(\frac{B}{R}\right)}^{2}\right], \end{array}$$

(8) $$\begin{array}{c} 8\pi T_{01}=-8\pi qAB=-2\left(\frac{{\dot{R}}^{\prime }}{R}-\frac{\dot{B}}{B}\frac{R^{\prime }}{R}-\frac{\dot{R}}{R}\frac{A^{\prime }}{A}\right), \end{array}$$

(9) $$\begin{array}{c} 8\pi T_{11}=8\pi P_{r}B^{2}=-{\left(\frac{B}{A}\right)}^{2}\left[2\frac{\ddot{R}}{R}-\left(2\frac{\dot{A}}{A}-\frac{\dot{R}}{R}\right)\frac{\dot{R}}{R}\right]+\left(2\frac{A^{\prime }}{A}+\frac{R^{\prime }}{R}\right)\frac{R^{\prime }}{R}-{\left(\frac{B}{R}\right)}^{2}, \end{array}$$

(10) $$\begin{array}{ccc} 8\pi T_{22} & = & \frac{8\pi }{\sin^{2}\theta }T_{33}=8\pi P_{⊥}R^{2}=-{\left(\frac{R}{A}\right)}^{2}\left[\frac{\ddot{B}}{B}+\frac{\ddot{R}}{R}-\frac{\dot{A}}{A}\left(\frac{\dot{B}}{B}+\frac{\dot{R}}{R}\right)+\frac{\dot{B}}{B}\frac{\dot{R}}{R}\right]\\ & + & {\left(\frac{R}{B}\right)}^{2}\left[\frac{A^{\prime \prime }}{A}+\frac{R^{\prime \prime }}{R}-\frac{A^{\prime }}{A}\frac{B^{\prime }}{B}+\left(\frac{A^{\prime }}{A}-\frac{B^{\prime }}{B}\right)\frac{R^{\prime }}{R}\right], \end{array}$$

where dots and primes denote derivatives with respect to *t* and *r*, respectively.

### 2.2. Kinematical Variables and the Mass Function

The three non-vanishing kinematical variables are the four-acceleration $a_{\alpha }$, the expansion scalar Θ and the shear tensor $\sigma_{\alpha \beta }$. The corresponding expressions follow at once from their definitions.

Thus,

(11) $$a_{\alpha }=V_{\alpha ;\beta }V^{\beta },$$

producing

(12) $$a_{1}=\frac{A^{\prime }}{A},\;\;a^{2}=a^{\alpha }a_{\alpha }={\left(\frac{A^{\prime }}{AB}\right)}^{2},$$

with $a^{\alpha }=a\chi^{\alpha }$.

The expansion Θ is given by

(13) $$\Theta ={V^{\alpha }}_{;\alpha }=\frac{1}{A}\left(\frac{\dot{B}}{B}+2\frac{\dot{R}}{R}\right),$$

and for the shear tensor, we have

(14) $$\sigma_{\alpha \beta }=V_{\left(\alpha ;\beta \right)}+a_{(\alpha }V_{\beta )}-\frac{1}{3}\Theta h_{\alpha \beta },$$

with only one non-vanishing independent component.

Using (5) and (14), we may write

(15) $$\sigma_{\alpha \beta }=\sigma \left(\chi_{\alpha }\chi_{\beta }-\frac{h_{\alpha \beta }}{3}\right),$$

where

(16) $$\sigma =\frac{1}{A}\left(\frac{\dot{B}}{B}-\frac{\dot{R}}{R}\right).$$

### 2.3. The Mass Function

Next, the mass function m(t,r) introduced by Misner and Sharp [20,21] is given by

(17) $$m\left(t,r\right)=\frac{R^{3}}{2}{R_{23}}^{23}=\frac{R}{2}\left[{\left(\frac{\dot{R}}{A}\right)}^{2}-{\left(\frac{R^{\prime }}{B}\right)}^{2}+1\right].$$

To study the dynamical properties of the system, let us introduce, following Misner and Sharp, the proper time derivative $D_{T}$, given by

(18) $$D_{T}=\frac{1}{A}\frac{\partial }{\partial t},$$

and the proper radial derivative $D_{R}$:

(19) $$D_{R}=\frac{1}{R^{\prime }}\frac{\partial }{\partial r}.$$

Using (18), we can define the velocity *U* of the collapsing fluid as the variation in the “areal” radius (*R*) with respect to the proper time, i.e.,

(20) $$U=D_{T}R.$$

Then, (17) can be rewritten as

(21) $$E\equiv \frac{R^{\prime }}{B}={\left[1+U^{2}-\frac{2m(t,r)}{R}\right]}^{1/2}.$$

From (17), we may easily obtain

(22) $$\begin{array}{c} D_{R}m=4\pi \left(\mu +q\frac{U}{E}\right)R^{2}. \end{array}$$

Equation (22) may be integrated to obtain

(23) $$m=\int_{0}^{r}4\pi R^{2}\left(\mu +q\frac{U}{E}\right)R^{\prime }dr,$$

(assuming a regular center for the distribution, so m(0)=0) or

(24) $$\frac{3m}{R^{3}}=4\pi \mu -\frac{4\pi }{R^{3}}\int_{0}^{r}R^{3}\mu^{\prime }dr+\frac{4\pi }{R^{3}}\int_{0}^{r}3q\frac{U}{E}R^{2}R^{\prime }dr.$$

### 2.4. The Junction Conditions

Outside $\Sigma^{\left(e\right)}$, we have the Vaidya spacetime (or the Schwarzschild spacetime in the dissipationless case), described by

(25) $$ds^{2}=-\left[1-\frac{2M(v)}{r}\right]dv^{2}-2drdv+r^{2}\left(d\theta^{2}+\sin^{2}\theta d\phi^{2}\right),$$

where M(v) denotes the total mass, and *v* is the retarded time. The matching of the non-adiabatic sphere to the Vaidya spacetime on the surface $r=r_{\Sigma^{\left(e\right)}}=$ constant, in the absence of thin shells (Darmois conditions [22]; see also [23]), implies the continuity of the first and second fundamental forms on the matching hypersurface, producing

(26) $$m\left(t,r\right)\overset{\Sigma^{\left(e\right)}}{=}M\left(v\right),$$

and

(27) $$q\overset{\Sigma^{\left(e\right)}}{=}P_{r}.$$

In a case when a cavity forms, we also have to match the solution to the Minkowski spacetime on the boundary surface delimiting the empty cavity ($\Sigma^{\left(i\right)}$). In this case, the matching conditions imply

(28) $$m\left(t,r\right)\overset{\Sigma^{\left(i\right)}}{=}0,$$

(29) $$q\overset{\Sigma^{\left(i\right)}}{=}P_{r}\overset{\Sigma^{\left(i\right)}}{=}0.$$

As we shall see below, in our model, the Darmois conditions cannot be satisfied on $\Sigma^{\left(i\right)}$, in which case we must allow for the presence of thin shells on $\Sigma^{\left(i\right)}$, implying discontinuities in the mass function [24].

On the other hand, Darmois conditions are satisfied on $\Sigma^{\left(e\right)}$ but only asymptotically (as t→∞). In other words, a thin shell is present on $\Sigma^{\left(e\right)}$ during the evolution, disappearing as the ghost star forms.

### 2.5. The Transport Equation

In the diffusion approximation, we shall need a transport equation to evaluate the temperature and its evolution within the fluid distribution. Here, we shall resort to using a transport equation derived from a causal dissipative theory (e.g., the Israel–Stewart second-order phenomenological theory for dissipative fluids [25,26,27]).

Thus, the corresponding transport equation for the heat flux reads as

(30) $$\tau h^{\alpha \beta }V^{\gamma }q_{\beta ;\gamma }+q^{\alpha }=-\kappa h^{\alpha \beta }\left(T_{,\beta }+Ta_{\beta }\right)-\frac{1}{2}\kappa T^{2}{\left(\frac{\tau V^{\beta }}{\kappa T^{2}}\right)}_{;\beta }q^{\alpha },$$

where κ denotes the thermal conductivity, and *T* and τ denote the temperature and relaxation time, respectively. Observe that, due to the symmetry of the problem, Equation (30) only has one independent component, which may be written as

(31) $$\tau \dot{q}=-\frac{1}{2}\kappa qT^{2}{\left(\frac{\tau }{\kappa T^{2}}\right)}^{˙}-\frac{1}{2}\tau q\Theta A-\frac{\kappa }{B}{\left(TA\right)}^{\prime }-qA.$$

In the case of τ=0, we recover the Eckart–Landau equation [28,29], and in the Newtonian limit, we recover the Cattaneo equation [30,31,32].

For simplicity, we shall consider here the so-called “truncated” version, where the last term in (30) is neglected [33]:

(32) $$\tau h^{\alpha \beta }V^{\gamma }q_{\beta ;\gamma }+q^{\alpha }=-\kappa h^{\alpha \beta }\left(T_{,\beta }+Ta_{\beta }\right),$$

whose only non-vanishing independent component becomes

(33) $$\tau \dot{q}+qA=-\frac{\kappa }{B}{\left(TA\right)}^{\prime }.$$

## 3. Three Conditions Underlying Our Model

As mentioned before, we shall start building up our model by imposing three conditions on the fluid distribution; these are the vanishing complexity factor condition, the quasi-homologous condition and a kinematical condition on the variation in the infinitesimal proper radial distance between two neighboring points per unit of proper time. In what follows, we shall briefly describe these conditions.

### 3.1. The Vanishing Complexity Factor Condition

The complexity factor is a scalar function that has been proposed in order to measure the degree of complexity of a given fluid distribution [11,12].

The complexity factor is identified with the scalar function $Y_{TF}$, which defines the trace-free part of the electric Riemann tensor (see [12] for details).

It can be expressed in terms of physical variables as

(34) $$Y_{TF}=-8\pi \Pi +\frac{4\pi }{R^{3}}\int_{0}^{r}R^{3}\left(\mu^{\prime }-\frac{3qBU}{R}\right)dr,$$

or in terms of the metric functions

(35) $$\begin{array}{c} Y_{TF}=\frac{1}{A^{2}}\left[\frac{\ddot{R}}{R}-\frac{\ddot{B}}{B}+\frac{\dot{A}}{A}\left(\frac{\dot{B}}{B}-\frac{\dot{R}}{R}\right)\right]+\frac{1}{B^{2}}\left[\frac{A^{\prime \prime }}{A}-\frac{A^{\prime }}{A}\left(\frac{B^{\prime }}{B}+\frac{R^{\prime }}{R}\right)\right]. \end{array}$$

We shall impose the vanishing of the complexity factor in order to find an analytical solution; however, as we shall see below, such a solution does not satisfy the required asymptotic behavior. In order to obtain a model with the appropriate asymptotic behavior, we shall modify this primeval solution, as a consequence of which the resulting model will satisfy the vanishing complexity factor condition only asymptotically.

### 3.2. The Quasi-Homologous Condition

The QH condition is a generalization of the homologous condition (H), which was assumed in [12] to represent the simplest mode of evolution of the fluid distribution. However, this last condition appears to be too stringent, thereby excluding many potentially interesting scenarios. Therefore, in [13], we proposed relaxing (H) and replaced it with what we called the “quasi–homologous” condition (QH).

More specifically, the H condition implies that

(36) $$U=\tilde{a}\left(t\right)R,\;\tilde{a}\left(t\right)\equiv \frac{U_{\Sigma^{\left(e\right)}}}{R_{\Sigma }^{\left(e\right)}},$$

and

(37) $$\frac{R_{I}}{R_{II}}=\mathrm{constant},$$

where $R_{I}$ and $R_{II}$ denote the areal radii of two concentric shells (I,II) described by $r=r_{I}=\mathrm{constant}$ and $r=r_{II}=\mathrm{constant}$, respectively.

These relationships are characteristic of homologous evolution in Newtonian hydrodynamics [15,16,17]. Moreover, in this latter case, (36) implies (37). However, in the relativistic case, both (36) and (37) are, in general, independent, and the former implies the latter only in very special cases.

On the other hand, the QH condition only requires (36), which, using the field equations, may also be written as (see [13] for details)

(38) $$\frac{4\pi }{R^{\prime }}Bq+\frac{\sigma }{R}=0.$$

As has already been mentioned, we shall start building up our model by assuming that the evolution of the fluid distribution proceeds in a quasi-homologous regime (QH). Since such a condition leads to asymptotic behavior which is incompatible with the formation of a ghost star, we should modify the primeval solution. As a consequence of this modification, the final solution will not satisfy the QH condition except in the static limit t→∞ when it is trivially satisfied.

### 3.3. A Kinematical Restriction

In order to obtain our primeval model, besides imposing the conditions of the vanishing complexity factor and quasi-homologous evolution, we shall impose a condition on a kinematical variable. To do this, let us first introduce another concept of velocity, different from *U*, which measures the variation in the infinitesimal proper radial distance between two neighboring points (δl) per unit of proper time, i.e., $D_{T}\left(\delta l\right)$. Thus, it can be shown that (see [14,34] for details)

(39) $$\frac{D_{T}\left(\delta l\right)}{\delta l}=\frac{1}{3}\left(2\sigma +\Theta \right),$$

or

(40) $$\frac{D_{T}\left(\delta l\right)}{\delta l}=\frac{\dot{B}}{AB}.$$

As an additional restriction, we assume that $D_{T}\left(\delta l\right)=0$, in which case B=B(r), from which a reparametrization of the coordinate *r* allows us to write, without a loss of generality, B=1, implying $R^{\prime }=E$, and from (13) and (16) it follows that

(41) $$\sigma =-\frac{U}{R}=-\frac{\Theta }{2}.$$

Since the center of symmetry (r=0) does not move throughout the evolution, it appears evident that any evolving fluid satisfying the condition B=1 cannot fill the central region. Therefore, we shall assume the center to be surrounded by a void cavity with the boundary surface $\Sigma^{\left(i\right)}$, whose areal radius changes in such a way that $D_{T}\left(\delta l\right)=0$ for all fluid elements.

From the comment above, it should be clear why this condition has been considered in the past as a useful tool for describing galactic voids. However, we should stress the fact that here we are adopting this kinematical condition as a heuristic hypothesis only in order to obtain an analytical model describing the emergence of a ghost star.

In the next section, we shall build up a model resulting in the depiction of a ghost star.

## 4. Building up the Model

We shall now proceed to construct a model giving rise to the depiction of a ghost star. This will be achieved in three steps. First, we shall find a primeval solution satisfying the three conditions $Y_{TF}=0$, (38) and B=1. In the second step, we shall modify this primeval solution in order to satisfy the desired asymptotic behavior. Finally, in the third step, we shall make a specific selection of some arbitrary functions and constants to fully determine the model. This final model will satisfy the condition B=1, but conditions $Y_{TF}=0$ and (38) will only be satisfied asymptotically.

### 4.1. The Primeval Solution

Let us start by considering a model satisfying the constraint B=1. This model is endowed with a cavity surrounding the center; accordingly, we should not worry about regularity conditions at the center.

In this case, the physical variables read as

(42) $$8\pi \mu =\frac{1}{A^{2}}\frac{{\dot{R}}^{2}}{R^{2}}-\frac{2R^{\prime \prime }}{R}-\frac{R^{\prime 2}}{R^{2}}+\frac{1}{R^{2}},$$

(43) $$8\pi P_{r}=-\frac{1}{A^{2}}\left(\frac{2\ddot{R}}{R}-\frac{2\dot{A}}{A}\frac{\dot{R}}{R}+\frac{{\dot{R}}^{2}}{R^{2}}\right)+\frac{2A^{\prime }}{A}\frac{R^{\prime }}{R}+\frac{{R^{\prime }}^{2}}{R^{2}}-\frac{1}{R^{2}},$$

(44) $$8\pi P_{⊥}=-\frac{1}{A^{2}}\left(\frac{\ddot{R}}{R}-\frac{\dot{A}}{A}\frac{\dot{R}}{R}\right)+\frac{A^{\prime \prime }}{A}+\frac{R^{\prime \prime }}{R}+\frac{A^{\prime }}{A}\frac{R^{\prime }}{R},$$

(45) $$4\pi q=\frac{1}{A}\left(\frac{{\dot{R}}^{\prime }}{R}-\frac{A^{\prime }}{A}\frac{\dot{R}}{R}\right)=-\sigma^{\prime }-\sigma \frac{R^{\prime }}{R},$$

and for the kinematical variables, we have

(46) $$\sigma =-\frac{\dot{R}}{AR},\;\Theta =\frac{2\dot{R}}{AR}.$$

Next, imposing the quasi-homologous condition, we obtain

(47) $$U=\tilde{a}\left(t\right)R\;\Rightarrow \;\tilde{a}\left(t\right)=\frac{\dot{R}}{AR}\;\Rightarrow \;\sigma =-\tilde{a}\left(t\right).$$

In other words, the QH condition implies that in this case, σ only depends on *t*.

On the other hand, the condition $Y_{TF}=0$ produces

(48) $$\begin{array}{ccc} Y_{TF} & = & \frac{1}{A^{2}}\left(\frac{\ddot{R}}{R}-\frac{\dot{A}}{A}\frac{\dot{R}}{R}\right)+\frac{A^{\prime \prime }}{A}-\frac{A^{\prime }}{A}\frac{R^{\prime }}{R} \end{array}$$

(49) $$\begin{array}{ccc} & = & \sigma^{2}-\frac{\dot{\sigma }}{A}+\frac{A^{\prime \prime }}{A}-\frac{A^{\prime }}{A}\frac{R^{\prime }}{R}=0. \end{array}$$

Thus, the conditions of a vanishing complexity factor, B=1 and quasi-homologous evolution read as

(50) $$A^{\prime \prime }-\frac{A^{\prime }R^{\prime }}{R}+A\sigma^{2}=\dot{\sigma },$$

and

(51) $$\frac{\dot{R}}{R}=-\sigma A,$$

respectively, with σ=σ(t).

In order to solve the above system of equations, it would be useful to introduce intermediate variables (X,Y):

(52) $$A=X+\frac{\dot{\sigma }}{\sigma^{2}}\;\mathrm{and}\;R=X^{\prime }Y,$$

in terms of which (50) and (51) become

(53) $$-\frac{X^{\prime }}{X}\frac{Y^{\prime }}{Y}+\sigma^{2}=0,$$

(54) $$\frac{{\dot{X}}^{\prime }}{X^{\prime }}+\frac{\dot{Y}}{Y}=-\sigma X-\frac{\dot{\sigma }}{\sigma }.$$

In what follows, we shall impose an additional restriction to solve the above system; specifically, we shall assume that *X* is a separable function, i.e.,

(55) $$X=\tilde{X}\left(r\right)T\left(t\right).$$

Then, feeding (55) back into (53) and taking the *t*-derivative, we obtain

(56) $$-\frac{\tilde{X^{\prime }}}{\tilde{X}}{\left(\frac{\dot{Y}}{Y}\right)}^{\prime }+2\sigma \dot{\sigma }=0.$$

Likewise, feeding (55) back into (54) and taking the *r*-derivative, we obtain

(57) $${\left(\frac{\dot{Y}}{Y}\right)}^{\prime }=-\sigma {\tilde{X}}^{\prime }T.$$

The combination of (56) and (57) produces

(58) $$\frac{{\tilde{X}}^{\prime 2}}{\tilde{X}}=-\frac{2\dot{\sigma }}{T}\equiv \beta^{2},$$

where β is a constant.

Then, from the integration of (58), we have

(59) $$\tilde{X}=\frac{{\left(\beta r+c_{1}\right)}^{2}}{4}\;\mathrm{and}\;T\left(t\right)=-\frac{2\dot{\sigma }}{\beta^{2}},$$

where $c_{1}$ is a constant of integration. Thus, the metric functions become

(60) $$\begin{array}{ccc} A & = & \frac{\dot{\sigma }}{2\beta^{2}\sigma^{2}}\left[2\beta^{2}-\sigma^{2}{\left(\beta r+c_{1}\right)}^{2}\right], \end{array}$$

(61) $$\begin{array}{ccc} R & = & F\left(t\right)g\left(r\right)\left(\beta r+c_{1}\right)e^{\frac{\sigma^{2}}{4\beta^{2}}{\left(\beta r+c_{1}\right)}^{2}}, \end{array}$$

where F(t) and g(r) are two arbitrary functions of their arguments. This is a generalized version of the solution exhibited in Sec. 7.2.1 in [13].

However, functions F(t) and g(r) are not completely arbitrary. Indeed, taking the *t*-derivative of (61) and feeding it back into (51), we obtain

(62) $$\frac{\dot{F}}{F}=-\frac{\dot{\sigma }}{\sigma },$$

producing

(63) $$F=\frac{c_{2}}{\sigma },$$

where $c_{2}$ is an arbitrary constant.

The above result implies that in the static limit (when σ=0), F→∞, resulting in R→∞ in that limit.

Still worse, from the above and (61), it follows that

(64) $$\dot{R}=-\frac{c_{2}\dot{\sigma }g\left(\beta r+c_{1}\right)}{\sigma^{2}}.$$

Thus, if we want our system to be static in the limit t→∞, we should demand that $\frac{\dot{\sigma }}{\sigma^{2}}\to 0$ as t→∞, but because of (60), such a condition would imply that A→0 as t→∞, which of course is unacceptable.

On the other hand, using (60) and (61), the vanishing complexity factor condition (50) reads as

(65) $$\frac{\dot{\sigma }g^{\prime }\left(\beta r+c_{1}\right)}{\beta g}=0,$$

implying g=constant. However, as we shall see below, we shall need g=g(r) in order to satisfy the matching conditions.

In other words, the metric functions (60) and (61), obtained from the QH condition and the vanishing complexity factor condition, are incompatible with the condition that the system tends asymptotically (as t→∞) to a static regime. On the other hand, in the case of B=1, the QH condition implies that the shear scalar is a function of *t* only, and therefore, in order to achieve static asymptotic behavior, σ should be function of both *t* and *r*.

### 4.2. The Asymptotic Conditions

In order to obtain the expected asymptotic behavior, we shall assume the same forms for metric functions (60) and (61) but replace σ with an arbitrary function of *t* (say, f(t)), such that in the limit t→∞,

(66) $$\begin{array}{c} F(t)\to \gamma =constant>0,\;f(t)\to 0,\\ \frac{\dot{f}}{f^{2}}\to constant>0, \end{array}$$

where *g* is not a constant.

Obviously, such metric functions do not satisfy (50), (51) and (65) in general (for any *t*), although they do satisfy such conditions in the limit t→∞.

From the comments above, we shall assume our metric variables read as

(67) $$\begin{array}{ccc} A & = & \frac{\dot{f}}{2\beta^{2}f^{2}}\left[2\beta^{2}-f^{2}{\left(\beta r+c_{1}\right)}^{2}\right], \end{array}$$

(68) $$\begin{array}{ccc} R & = & F\left(t\right)g\left(r\right)\left(\beta r+c_{1}\right)e^{\frac{f^{2}}{4\beta^{2}}{\left(\beta r+c_{1}\right)}^{2}}. \end{array}$$

Using these expressions in (17) and (42)–(45), for the physical variables, we find

(69) $$\begin{array}{c} 8\pi \mu =\frac{4\beta^{4}f^{4}}{{\dot{f}}^{2}{\left[2\beta^{2}-f^{2}{\left(\beta r+c_{1}\right)}^{2}\right]}^{2}}{\left[\frac{\dot{F}}{F}+\frac{f\dot{f}{\left(\beta r+c_{1}\right)}^{2}}{2\beta^{2}}\right]}^{2}-2\left[\frac{g^{\prime \prime }}{g}+\frac{g^{\prime }\left[2\beta^{2}+f^{2}{\left(\beta r+c_{1}\right)}^{2}\right]}{g\beta (\beta r+c_{1})}\right.\\ \left.+\frac{f^{4}{\left(\beta r+c_{1}\right)}^{2}}{4\beta^{2}}+\frac{3f^{2}}{2}\right]-{\left[\frac{g^{\prime }}{g}+\frac{\beta }{\left(\beta r+c_{1}\right)}+\frac{f^{2}\left(\beta r+c_{1}\right)}{2\beta }\right]}^{2}+\frac{e^{\frac{-f^{2}{\left(\beta r+c_{1}\right)}^{2}}{2\beta^{2}}}}{F^{2}g^{2}{\left(\beta r+c_{1}\right)}^{2}} \end{array}$$

(70) $$\begin{array}{c} 8\pi P_{r}=-\frac{4\beta^{4}f^{4}}{{\dot{f}}^{2}{\left[2\beta^{2}-f^{2}{\left(\beta r+c_{1}\right)}^{2}\right]}^{2}}\left\{\frac{2\ddot{F}}{F}+\frac{2\dot{F}f\dot{f}{\left(\beta r+c_{1}\right)}^{2}}{F\beta^{2}}+\frac{{\left(\beta r+c_{1}\right)}^{2}\left({\dot{f}}^{2}+f\ddot{f}\right)}{\beta^{2}}\right.\\ \left.+\frac{{\dot{f}}^{2}f^{2}{\left(\beta r+c_{1}\right)}^{4}}{2\beta^{4}}-2\left[\frac{\ddot{f}}{\dot{f}}-\frac{4\beta^{2}\dot{f}}{f[2\beta^{2}-f^{2}{\left(\beta r+c_{1}\right)}^{2}]}\right]\left[\frac{\dot{F}}{F}+\frac{f\dot{f}{\left(\beta r+c_{1}\right)}^{2}}{2\beta^{2}}\right]\right.\\ \left.+{\left[\frac{\dot{F}}{F}+\frac{f\dot{f}{\left(\beta r+c_{1}\right)}^{2}}{2\beta^{2}}\right]}^{2}\right\}-\frac{e^{\frac{-f^{2}{\left(\beta r+c_{1}\right)}^{2}}{2\beta^{2}}}}{F^{2}g^{2}{\left(\beta r+c_{1}\right)}^{2}}-\frac{4\beta f^{2}\left(\beta r+c_{1}\right)}{\left[2\beta^{2}-f^{2}{\left(\beta r+c_{1}\right)}^{2}\right]}\left[\frac{g^{\prime }}{g}+\frac{\beta }{\left(\beta r+c_{1}\right)}+\frac{f^{2}\left(\beta r+c_{1}\right)}{2\beta }\right]\\ +{\left[\frac{g^{\prime }}{g}+\frac{\beta }{\left(\beta r+c_{1}\right)}+\frac{f^{2}\left(\beta r+c_{1}\right)}{2\beta }\right]}^{2}\;\; \end{array}$$

(71) $$\begin{array}{c} 8\pi P_{⊥}=-\frac{4\beta^{4}f^{4}}{{\dot{f}}^{2}{\left[2\beta^{2}-f^{2}{\left(\beta r+c_{1}\right)}^{2}\right]}^{2}}\left\{\frac{\ddot{F}}{F}+\frac{\dot{F}f\dot{f}{\left(\beta r+c_{1}\right)}^{2}}{F\beta^{2}}+\frac{{\left(\beta r+c_{1}\right)}^{2}\left({\dot{f}}^{2}+f\ddot{f}\right)}{2\beta^{2}}\right.\\ \left.+\frac{{\dot{f}}^{2}f^{2}{\left(\beta r+c_{1}\right)}^{4}}{4\beta^{4}}-\left[\frac{\ddot{f}}{\dot{f}}-\frac{4\beta^{2}\dot{f}}{f[2\beta^{2}-f^{2}{\left(\beta r+c_{1}\right)}^{2}]}\right]\left[\frac{\dot{F}}{F}+\frac{f\dot{f}{\left(\beta r+c_{1}\right)}^{2}}{2\beta^{2}}\right]\right\}\\ +\frac{g^{\prime \prime }}{g}-\frac{2\beta^{2}f^{2}}{\left[2\beta^{2}-f^{2}{\left(\beta r+c_{1}\right)}^{2}\right]}+\frac{f^{4}{\left(\beta r+c_{1}\right)}^{2}}{4\beta^{2}}+\frac{g^{\prime }\left[2\beta^{2}+f^{2}{\left(\beta r+c_{1}\right)}^{2}\right]}{g\beta (\beta r+c_{1})}-\\ \frac{2\beta f^{2}\left(\beta r+c_{1}\right)}{\left[2\beta^{2}-f^{2}{\left(\beta r+c_{1}\right)}^{2}\right]}\left[\frac{g^{\prime }}{g}+\frac{\beta }{\left(\beta r+c_{1}\right)}+\frac{f^{2}\left(\beta r+c_{1}\right)}{2\beta }\right]+\frac{3f^{2}}{2} \end{array}$$

(72) $$\begin{array}{c} 4\pi q=\frac{2\beta^{2}f^{2}}{\dot{f}\left[2\beta^{2}-f^{2}{\left(\beta r+c_{1}\right)}^{2}\right]}\left\{\frac{\dot{F}}{F}\left[\frac{g^{\prime }}{g}+\frac{\beta }{\left(\beta r+c_{1}\right)}+\frac{f^{2}\left(\beta r+c_{1}\right)}{2\beta }\right]+\frac{g^{\prime }f\dot{f}{\left(\beta r+c_{1}\right)}^{2}}{2\beta^{2}g}\right.\\ \left.+\frac{f\dot{f}\left(\beta r+c_{1}\right)}{2\beta }\left[3+\frac{f^{2}{\left(\beta r+c_{1}\right)}^{2}}{2\beta^{2}}\right]+\frac{2\beta f^{2}\left(\beta r+c_{1}\right)}{\left[2\beta^{2}-f^{2}{\left(\beta r+c_{1}\right)}^{2}\right]}\left[\frac{\dot{F}}{F}+\frac{f\dot{f}{\left(\beta r+c_{1}\right)}^{2}}{2\beta^{2}}\right]\right\} \end{array}$$

(73) $$\begin{array}{c} m=\frac{R}{2}\left\{1+\frac{4R^{2}\beta^{4}f^{4}}{{\dot{f}}^{2}{\left[2\beta^{2}-f^{2}{\left(\beta r+c_{1}\right)}^{2}\right]}^{2}}{\left[\frac{\dot{F}}{F}+\frac{f\dot{f}{\left(\beta r+c_{1}\right)}^{2}}{2\beta^{2}}\right]}^{2}\right.\\ \left.-R^{2}{\left[\frac{g^{\prime }}{g}+\frac{\beta }{\left(\beta r+c_{1}\right)}+\frac{f^{2}\left(\beta r+c_{1}\right)}{2\beta }\right]}^{2}\right\}. \end{array}$$

### 4.3. The Matching Conditions

So far, our model is determined up to three functions, F(t),f(t) and g(r). The form of these functions will be suggested by the asymptotic conditions as t→∞ and a condition to avoid shell-crossing singularities ($R^{\prime }>0$).

We are looking for a model which asymptotically (as t→∞) approaches the state of a static ghost star, $m(t\to \infty ,r_{\Sigma^{\left(e\right)}})=0$.

As mentioned before, to achieve the asymptotic behavior required of the model, we must demand that in the limit t→∞, the conditions in (66) are satisfied.

Let us now consider the matching of this model on $\Sigma^{\left(e\right)}$. We shall demand that the matching conditions (26) and (27) be satisfied asymptotically (as t→∞) when a ghost star is expected to form. Thus, we shall demand that

(74) $$m(\infty ,r_{\Sigma^{\left(e\right)}})=0.$$

On the other hand, as can be seen from (72) and (66), in the limit t→∞, we obtain q→0 as expected from the static limit; therefore, we must also demand that

(75) $$P_{r}\left(\infty ,r_{\Sigma^{\left(e\right)}}\right)=0.$$

Using (66) in (73), the condition $m(t\to \infty ,r_{\Sigma^{\left(e\right)}})=0$ reads as

(76) $$\gamma g_{\Sigma^{\left(e\right)}}\left(\beta r_{\Sigma^{\left(e\right)}}+c_{1}\right)\left[\frac{g_{\Sigma^{\left(e\right)}}^{\prime }}{g_{\Sigma^{\left(e\right)}}}+\frac{\beta }{\left(\beta r_{\Sigma^{\left(e\right)}}+c_{1}\right)}\right]=1.$$

To specify our model further, we shall assume that for the function g(r) and the constant $c_{1}$,

(77) $$g=c_{3}r,\;c_{1}=0,$$

where $c_{3}$ is a dimensionless constant.

Then, condition (76) becomes

(78) $$r_{\Sigma^{\left(e\right)}}=\frac{1}{2\gamma c_{3}\beta }.$$

On the other hand, condition (75) reads as

(79) $$\frac{g_{\Sigma^{\left(e\right)}}^{\prime }}{g_{\Sigma^{\left(e\right)}}}+\frac{1}{r_{\Sigma^{\left(e\right)}}}=\frac{1}{\gamma g_{\Sigma^{\left(e\right)}}\beta r_{\Sigma^{\left(e\right)}}},$$

where (66) and $c_{1}=0$ have been used. Feeding (77) back into (79), we obtain

(80) $$r_{\Sigma^{\left(e\right)}}=\frac{1}{2\gamma c_{3}\beta },$$

which is exactly (78). Thus, the above choice of constants ensures the asymptotic fulfillment of matching conditions on $\Sigma^{\left(e\right)}$ for our fluid distribution with Minkowski spacetime.

It is worth mentioning that for this choice of the values of *g* and $c_{1}$, the matching conditions are not satisfied on $\Sigma^{\left(i\right)}$. Therefore, this model has a thin shell on this surface, and $r_{\Sigma^{\left(i\right)}}$ is a free parameter.

### 4.4. The Model

Finally, in order to fully describe our model, we have to specify the two functions, *F* and *f*, which must satisfy the asymptotic conditions in (66).

For the sake of simplicity, we choose

(81) $$F=\gamma e^{-\frac{r_{\Sigma^{\left(e\right)}}}{t}},\;f=-\frac{1}{t}.$$

With the above choice and (77) and (80), the metric functions *A* and *R* read as

(82) $$A=1-\frac{x^{2}}{2t^{*2}},\;R=\frac{r_{\Sigma^{\left(e\right)}}}{2}e^{-1/t^{*}}x^{2}e^{\frac{x^{2}}{4t^{*2}}},$$

where $t^{*}\equiv \frac{t}{r_{\Sigma^{\left(e\right)}}}$ changes in the interval $\left[\frac{t_{0}}{r_{\Sigma^{\left(e\right)}}},\infty \right]$, with $t_{0}$ being a positive constant, and $x\equiv \frac{r}{r_{\Sigma^{\left(e\right)}}}$ changes in the interval $\left[\frac{r_{\Sigma^{\left(i\right)}}}{r_{\Sigma^{\left(e\right)}}},1\right]$.

In order to ensure the positivity of *A*, we must assume that $t^{*}>\frac{1}{\sqrt{2}}$.

Using the above expressions in (69)–(73), the physical variables describing our model read as

(83) $$8\pi \mu =\frac{4t^{*4}{\left(\frac{1}{t^{*2}}-\frac{x^{2}}{2t^{*3}}\right)}^{2}}{r_{\Sigma^{\left(e\right)}}^{2}{\left(2t^{*2}-x^{2}\right)}^{2}}-\frac{2}{r_{\Sigma^{\left(e\right)}}^{2}}\left[\frac{\left(2t^{*2}+x^{2}\right)}{t^{*2}x^{2}}+\frac{x^{2}}{4t^{*4}}+\frac{3}{2t^{*2}}\right]-\frac{1}{r_{\Sigma^{\left(e\right)}}^{2}}{\left(\frac{2}{x}+\frac{x}{2t^{*2}}\right)}^{2}+\frac{4e^{\left(\frac{2}{t^{*}}-\frac{x^{2}}{2t^{*2}}\right)}}{r_{\Sigma^{\left(e\right)}}^{2}x^{4}},$$

(84) $$\begin{array}{c} 8\pi P_{r}=-\frac{4t^{*4}}{r_{\Sigma^{\left(e\right)}}^{2}{\left(2t^{*2}-x^{2}\right)}^{2}}\left\{2\left(\frac{1}{t^{*4}}-\frac{2}{t^{*3}}\right)-\frac{2x^{2}}{t^{*5}}+\frac{3x^{2}}{t^{*4}}+\frac{x^{4}}{2t^{*6}}\right.\\ \left.-2\left[-\frac{2}{t^{*}}+\frac{4t^{*}}{\left(2t^{*2}-x^{2}\right)}\right]\left(\frac{1}{t^{*2}}-\frac{x^{2}}{2t^{*3}}\right)+{\left(\frac{1}{t^{*2}}-\frac{x^{2}}{2t^{*3}}\right)}^{2}\right\}\\ -\frac{4e^{\left(\frac{2}{t^{*}}-\frac{x^{2}}{2t^{*2}}\right)}}{r_{\Sigma^{\left(e\right)}}^{2}x^{4}}-\frac{4x}{r_{\Sigma^{\left(e\right)}}^{2}\left(2t^{*2}-x^{2}\right)}\left(\frac{2}{x}+\frac{x}{2t^{*2}}\right)+\frac{{\left(\frac{2}{x}+\frac{x}{2t^{*2}}\right)}^{2}}{r_{\Sigma^{\left(e\right)}}^{2}}, \end{array}$$

(85) $$\begin{array}{c} 8\pi P_{⊥}=-\frac{4t^{*4}}{r_{\Sigma^{\left(e\right)}}^{2}{\left(2t^{*2}-x^{2}\right)}^{2}}\left\{\left(\frac{1}{t^{*4}}-\frac{2}{t^{*3}}\right)-\frac{x^{2}}{t^{*5}}+\frac{x^{4}}{4t^{*6}}-\left[-\frac{2}{t^{*}}+\frac{4t^{*}}{\left(2t^{*2}-x^{2}\right)}\right]\left(\frac{1}{t^{*2}}-\frac{x^{2}}{2t^{*3}}\right)\right.\\ \left.+\frac{3x^{2}}{2t^{*4}}\right\}-\frac{2}{r_{\Sigma^{\left(e\right)}}^{2}\left(2t^{*2}-x^{2}\right)}-\frac{2x}{r_{\Sigma^{\left(e\right)}}^{2}\left(2t^{*2}-x^{2}\right)}\left(\frac{2}{x}+\frac{x}{2t^{*2}}\right)+\frac{x^{2}}{r_{\Sigma^{\left(e\right)}}^{2}4t^{*4}}\\ +\frac{\left(2t^{*2}+x^{2}\right)}{r_{\Sigma^{\left(e\right)}}^{2}x^{2}t^{*2}}+\frac{3}{2r_{\Sigma^{\left(e\right)}}^{2}t^{*2}}, \end{array}$$

(86) $$4\pi q=\frac{2}{r_{\Sigma^{\left(e\right)}}^{2}\left(2t^{*2}-x^{2}\right)}\left[\frac{2}{x}+\frac{x}{2t^{*2}}-\frac{2x}{t^{*}}-\frac{x^{3}}{4t^{3*}}+\frac{x(2t^{*}-x^{2})}{t^{*}\left(2t^{*2}-x^{2}\right)}\right],$$

(87) $$m=\frac{r_{\Sigma^{\left(e\right)}}x^{2}e^{-\frac{1}{t^{*}}}e^{\frac{x^{2}}{4t^{*2}}}}{4}\left\{1+\frac{x^{4}e^{-\frac{2}{t^{*}}}e^{\frac{x^{2}}{2t^{*2}}}}{4}\left[\frac{{\left(2t*-x^{2}\right)}^{2}}{t^{*2}{\left(2t^{*2}-x^{2}\right)}^{2}}-\frac{{\left(4t^{*2}+x^{2}\right)}^{2}}{4x^{2}t^{*4}}\right]\right\}.$$

The temperature for this model may be calculated using (33), producing

(88) $$T^{*}=-\frac{1}{4\pi }\int (\tau^{*}\frac{\partial q^{*}}{\partial t^{*}}+q^{*}A)dx+\Phi \left(t\right),$$

where $T^{*}\equiv \kappa Tr_{\Sigma^{\left(e\right)}}$, $\tau^{*}\equiv \frac{\tau }{r_{\Sigma^{\left(e\right)}}}$, $q^{*}\equiv 4\pi qr_{\Sigma^{\left(e\right)}}^{2}$, Φ(t) is an arbitrary function of integration and *A* and *q* are given by (82) and (86), respectively.

However, the resulting expression is cumbersome and not very illuminating. Worse still, it depends on an arbitrary function (Φ) and the numerical value of the relaxation time τ. The former may be related to the temperature at the boundary surface, but this is also unknown unless we specify the microphysics of the fluid further. On the other hand, the numerical value of the relaxation time also depends on the microphysics of the fluid. However, a microscopic setup of the model is out of the scope of this work.

Thus, the only way to obtain the required information is by assigning the value of τ and the profile of Φ in an ad hoc way, which, due to its intrinsic arbitrariness, deprives the obtained expression for the temperature of any physical relevance. Accordingly, we will dispense with the temperature graphic. It is suffice to say that asymptotically, the temperature tends to a constant, as expected from a static distribution in thermal equilibrium (as we shall see below, the “thermal inertial term” Ta [35] vanishes asymptotically in this model).

We shall now illustrate the formation of the ghost star as t→∞ for the model described so far. To do that, we need to evaluate the energy density in the limit t→∞. Integrating (66), (77) and (80) in (69), we obtain

(89) $$8\pi \mu \left(t\to \infty ,r\right)=\frac{4}{r_{\Sigma^{\left(e\right)}}^{2}}\left(\frac{1-2x^{2}}{x^{4}}\right),$$

where $x\equiv \frac{r}{r_{\Sigma^{\left(e\right)}}}$, whose values are within the interval $\left[\frac{r_{\Sigma^{\left(i\right)}}}{r_{\Sigma^{\left(e\right)}}},1\right]$.

With the choices made above, the expressions for $U_{\Sigma^{\left(e\right)}}$ and $m_{\Sigma^{\left(e\right)}}$ read as

(90) $$U_{\Sigma^{\left(e\right)}}=\frac{(2t^{*}-1)\Psi }{2t^{*}\left(2t^{2*}-1\right)},$$

and

(91) $$m_{\Sigma^{\left(e\right)}}=\frac{\Psi r_{\Sigma^{\left(e\right)}}}{4}\left[1+\frac{\Psi^{2}{\left(1-\frac{1}{2t^{*}}\right)}^{2}}{t^{*4}{\left(2-\frac{1}{t^{2*}}\right)}^{2}}-\frac{\Psi^{2}}{4}{\left(2+\frac{1}{2t^{*2}}\right)}^{2}\right],$$

where $\Psi \equiv e^{-\frac{1}{t^{*}}+\frac{1}{4t^{*2}}}$.

**Figure 1:**
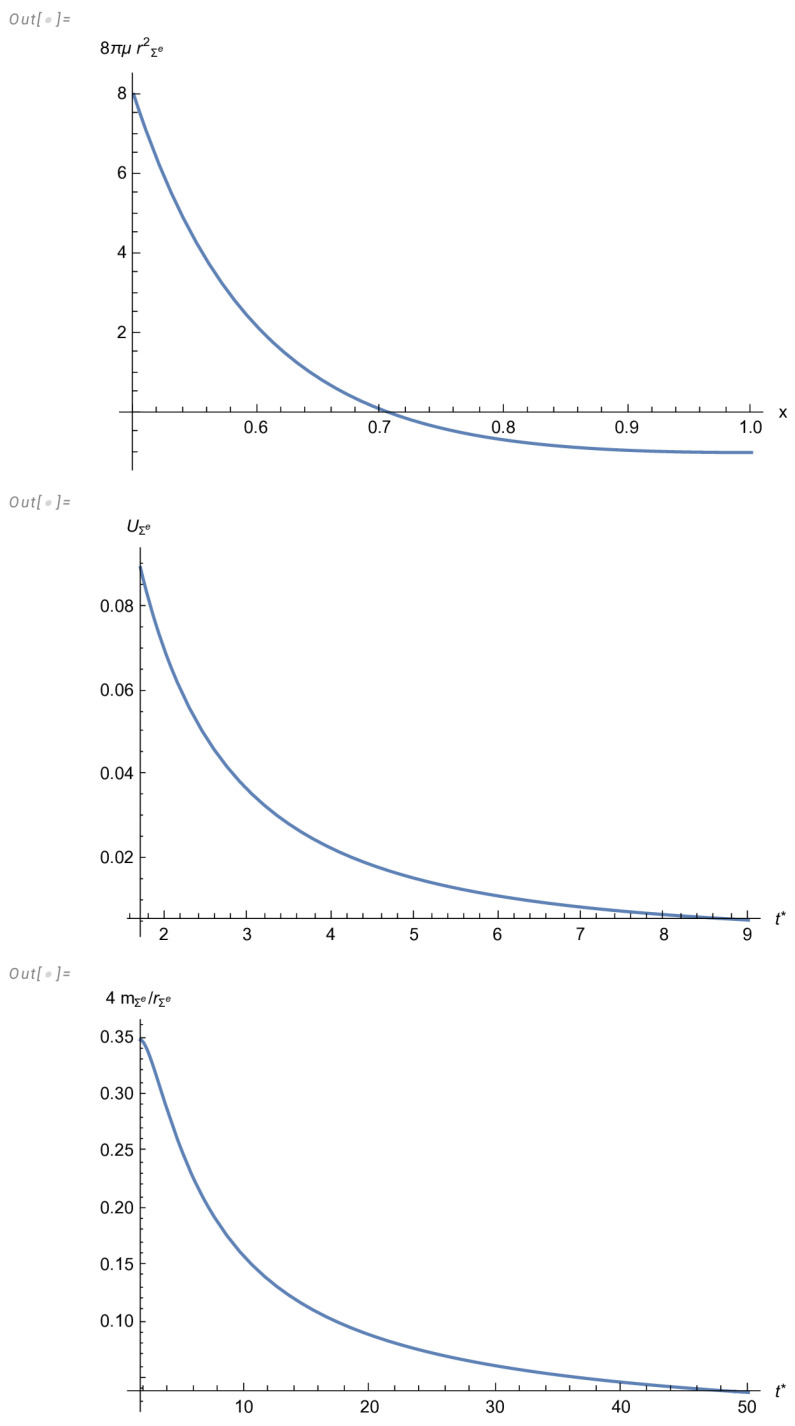
$8\pi \mu r_{\Sigma^{\left(e\right)}}^{2}$, evaluated at $t^{*}\to \infty$, as function of *x* in the interval $\left[\frac{1}{2},1\right]$; $U_{\Sigma^{\left(e\right)}}$ and $m_{\Sigma^{\left(e\right)}}$ as functions of $t^{*}$.

The three curves in Figure 1 illustrate the emergence of the ghost star. The first curve depicts the radial distribution of the energy density as t→∞ and shows a region of negative values for this variable, which ensures the vanishing of the total mass, as illustrated by the third curve. Finally, the second curve shows the tendency to a static situation.

**Figure 2:**
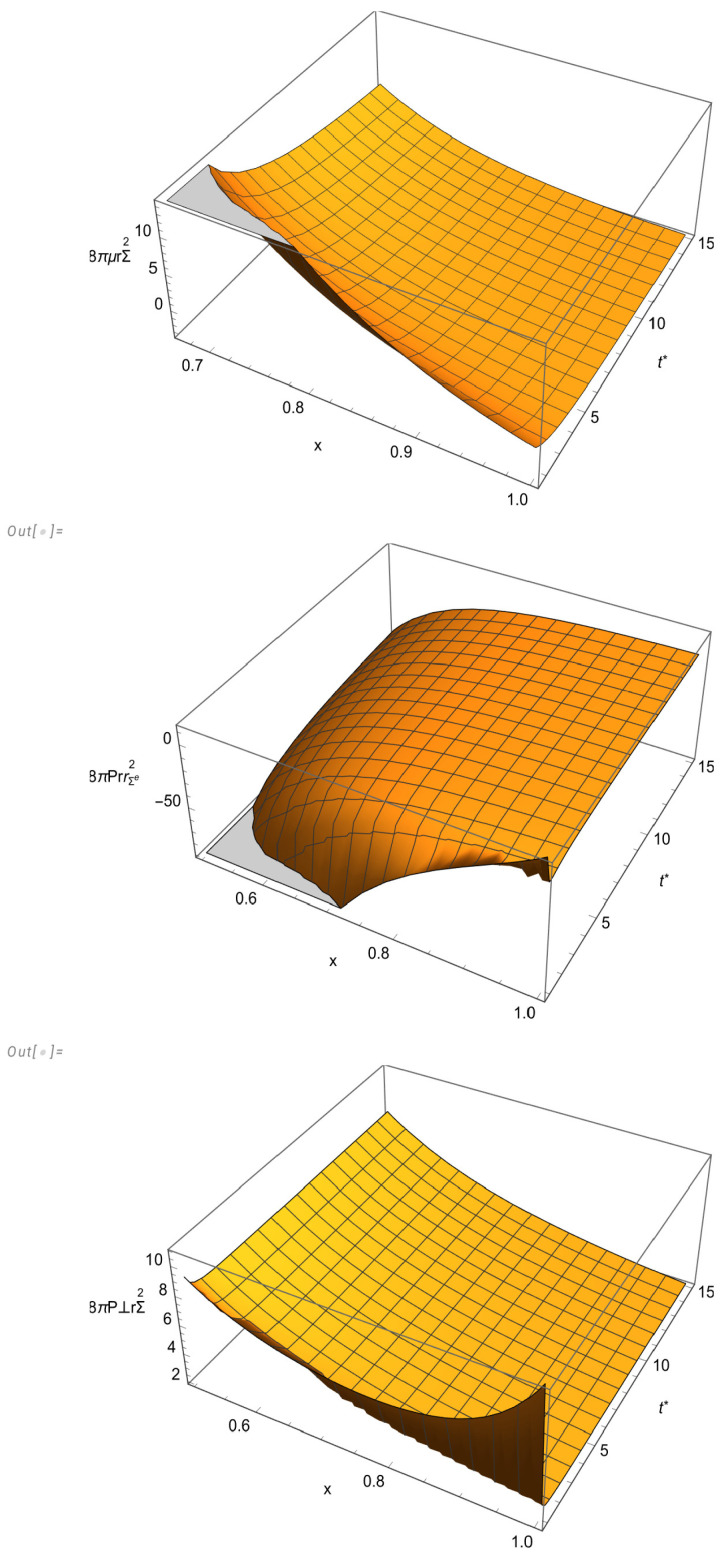
$8\pi \mu r_{\Sigma^{\left(e\right)}}^{2}$, $8\pi P_{r}r_{\Sigma^{\left(e\right)}}^{2}$ and $8\pi P_{⊥}r_{\Sigma^{\left(e\right)}}^{2}$ as functions of *x* and $t^{*}$.

**Figure 3:**
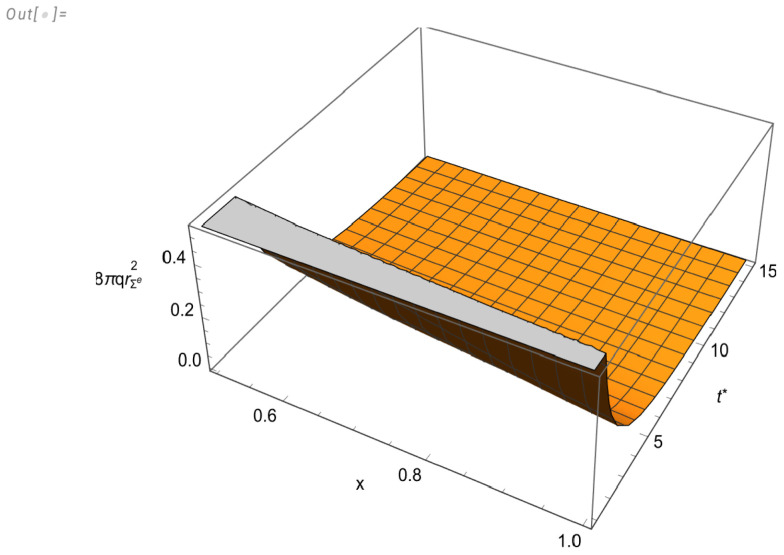
$8\pi qr_{\Sigma^{\left(e\right)}}^{2}$ as function of *x* and $t^{*}$.

The behavior of the physical variables is depicted in Figure 2 and Figure 3. The graphics for $P_{r},P_{⊥}$ and *q* were drawn for the variables $t^{*}$ and *x* in the intervals [0.9,15] and [0.5,1], respectively. On the other hand, for μ, we chose the intervals [1.7,15] and [0.67,1] in order to better illustrate the appearance of the region of a negative energy density. The fast convergence of the system to the static regime is well illustrated in Figure 3.

## 5. Discussion

The main purpose of this work has been to exhibit the viability of the formation of a ghost star as the end point of the evolution of a self-gravitating fluid distribution. To achieve this goal, we have presented an analytical model of a dissipative spherically symmetric fluid distribution evolving toward a ghost star.

We initially found a primeval solution satisfying the vanishing complexity factor condition, quasi-homologous evolution and B=1. Next, this primeval solution was modified to satisfy the required asymptotic conditions in (66). Finally, we chose the remaining arbitrary functions to fully specify our model. This final model represents an expanding fluid distribution endowed with a cavity surrounding the center, tending to a static configuration. The end point of the evolution of this model is a ghost star, as illustrated by Figure 1.

Furthermore, in the limit t→∞, we have A→1, implying, because of (12), the vanishing of the four-acceleration of the fluid forming the ghost star (which explains the vanishing of the “thermal inertial term” mentioned before). This implies, in turn, according to (A1), that the gravitational term in the dynamic Equation (A2) (the Tolman mass [36]) vanishes and the equilibrium is reached through a balance between the radial pressure gradient and the anisotropic factor. A particular model of a ghost star with a vanishing complexity factor and vanishing Tolman mass was considered in [1].

The model satisfies asymptotic Darmois conditions on the external boundary surface, whereas on the inner boundary surface, such conditions are not satisfied, thereby indicating the appearance of shells on this hypersurface. The presence of these thin shells is likely to be a result of the simplicity of the model. More involved analytical models or numerical models could avoid these “drawbacks”.

We should recall that the very existence of ghost stars relies on the assumption of the existence of regions of a fluid distribution endowed with a negative energy density. In this respect, it should be mentioned that a negative energy density (or negative mass) is a subject extensively considered in the literature (see [37,38,39,40,41,42,43,44,45,46,47,48,49,50,51,52] and the references therein). Particularly relevant are those references relating the appearance of a negative energy density to quantum effects.

It is worth mentioning the relevance of the observational aspects of ghost stars, in general and of our model in particular. On the one hand, it is evident that the shadow of this kind of object should differ from the one produced by a self-gravitating star with a non-vanishing total mass. In the particular case of the model considered here, one should be able to detect the variation in the shadow as the system approaches the state of a ghost star. We have not examined if this has been achieved in ongoing observations of this kind [53,54,55,56], but this is an important issue to consider. A research endeavor in a similar direction has recently been published in [57].

In the same order of ideas, it should be clear that the radiation emitted from the surface of a ghost star should not exhibit gravitational redshift, opening the way for the possible detection of such objects. In our case, a continuous measurement of such redshift and its ensuing decrease as evolution progresses would indicate the formation of a ghost star.

On the other hand, it is worth noting that ghost stars are a sort of reservoir of dark mass produced by the appearance of a negative energy density is some regions of a fluid distribution. It remains to be seen if the general problem of dark matter could, at least partially, be explained in terms of ghost stars [58].

We would also like to mention that an important piece of theoretical evidence supporting the concept of a ghost star is still missing. We have in mind a microscopic theory accounting for the appearance of a negative energy density. Research in this direction could provide further support for the astrophysical relevance of ghost stars by allowing us to clarify important questions about the structure of these objects, such as their stability.

Finally, let us mention two natural extensions of the work presented here:

- Our solution was based on a set of heuristic conditions mentioned above. Alternatively, solutions of this kind might be found by using the general methods presented in [59,60,61,62] or utilizing some of the recently presented results in the study of gravitational collapse (see, for example, [63,64,65] and the references therein).
- We have resorted to using GR to describe the gravitational interaction. It would be interesting to consider the same problem within the context of one of the extended gravitational theories [66].

## Data Availability

Data are contained within the article.

## Appendix A. Dynamical Equation

Using (12), (17), (20) and (70), we can easily obtain

(A1) $$D_{T}U=-\frac{m}{R^{2}}-4\pi P_{r}R,+Ea,$$

which allows us to write the dynamical equation following from the Bianchi identities as (see [12] for details)

(A2) $$\begin{array}{c} \left(\mu +P_{r}\right)D_{T}U=-\left(\mu +P_{r}\right)\left[\frac{m}{R^{2}}+4\pi P_{r}R\right]\\ -E^{2}\left[D_{R}P_{r}+2\left(P_{r}-P_{⊥}\right)\frac{1}{R}\right]-E\left[D_{T}q+2q\left(2\frac{U}{R}+\sigma \right)\right]. \end{array}$$
